# Supplementary material for: Neglected Fractures of the Lateral Humeral Condyle in Children; Which Treatment for Which Condition?
Source: Children (Basel). 2021 Jan 18;8(1):56. doi: 10.3390/children8010056 (PMC7830377; doi:10.3390/children8010056)
Supplement: Supplementary file 1 [file children-08-00056-s001.zip › table 1 Suppl..docx]

| **PATIENT** | **DEMOGRAPHICS**  **SEX/SIDE/AGE (years)** | **INJURY DURATION (months)** | **FRACTURE GRADING MILCH/WEISS/SONG** | **INITIAL TREATMENT** | **DISPLACEMENT (mm)**  **LATERAL-MEDIAL** | **TREATMENT** | **BONE GRAFT** | **HARDWARE** | **CARRYING-ANGLE (degrees)** | **FLEXION-EXTENSION (degrees)** | **DHILLON SCORE OVERALL-FUNCTION** | **MEPS** |
| --- | --- | --- | --- | --- | --- | --- | --- | --- | --- | --- | --- | --- |
| 1 | M/R7.3 | 1.5 | 2/3/3 | CONSERVATIVE | 3-11 | CONSERVATIVE | - | - | 0 | 30-120 | 6-4 | 70 |
| 2 | M/R/4.5 | 2 | 2/2/3 | CONSERVATIVE | 4-5 | CONSERVATIVE | - | - | 7 | 40-110 | 5-2 | 30 |
| 3 | F/L/5.7 | 1.5 | 2/2/3 | CONSERVATIVE | 4-2 | CONSERVATIVE | - | - | NA | 50-120 | 2-2 | 55 |
| 4 | F/R/3.7 | 1.6 | 2/2/3 | CONSERVATIVE | 5-5 | CONSERVATIVE | - | - | NA | 40-140 | 6-4 | 70 |
| 5 | M/L/4.2 | 1.3 | 2/2/3 | CONSERVATIVE | 3-3 | CONSERVATIVE | - | - | NA | 45-90 | 3-0 | 30 |
| 6 | M/L/3.7 | 3 | 2/3/4 | CONSERVATIVE | 7-16 | CONSERVATIVE | - | - | 7 | 0-140 | 9-6 | 95 |
| 7 | F/R/4 | 2 | 2/3/3 | CONSERVATIVE | 4-7 | ISF | NO | 2 SCREWS | NA | 100-140 | 2-0 | 35 |
| 8 | M/R/3.4 | 4 | 2/2/3 | SURGICAL | 5-7 | ISF | YES (ALLOGRAFT) | 1 SCREW + 1 K-WIRE | NA | 40-145 | 6-4 | 70 |
| 9 | M/L/5.3 | 3 | 2/2/3 | CONSERVATIVE | 4-4 | ISF | NO | 2 SCREWS | NA | 40-145 | 6-4 | 70 |
| 10 | M/R/6.4 | 45 | 2/2/3 | SURGICAL | 3-4 | ISF | NO | 2 SCREWS | -35 | 10-140 | 5-5 | 85 |
| 11 | M/R/1.7 | 4 | 2/3/3 | CONSERVATIVE | 6-13 | ISF | NO | 2 K-WIRES | 7 | 0-145 | 7-4 | 50 |
| 12 | M/L/8.1 | 2 | 2/3/3 | CONSERVATIVE | 5-8 | ISF | NO | 1 SCREW | 10 | 35-140 | 6-3 | 80 |
| 13 | M/L/6.3 | 5 | 2/2/3 | CONSERVATIVE | 3-5 | ISF | YES | 1 SCREW | 7 | 0-135 | 7-4 | 70 |
| 14 | M/R/7.9 | 7 | 2/3/4 | CONSERVATIVE | 9-9 | ORIF | YES | 1 SCREW + 1 K-WIRE | 25 | 10-140 | 3-2 | 60 |
| 15 | M/R/4.1 | 17 | 2/3/5 | SURGICAL | 5-8 | ORIF | NO | 3 K-WIRES | NA | 40-90 | 5-4 | 70 |
| 16 | M/L/5.7 | 14 | 2/2/3 | CONSERVATIVE | 8-11 | ORIF | NO | 2 SCREWS | -30 | 0-145 | 6-6 | 100 |
| 17 | M/R/4.8 | 1 | 1/2/3 | CONSERVATIVE | 6-4 | ORIF | NO | 3 K-WIRES | NA | 70-90 | 3-3 | 30 |
| 18 | F/L/3.9 | 21 | 1/2/3 | CONSERVATIVE | 7-4 | ORIF | NO | 1 SCREW + 1 K-WIRE | 7 | 0-140 | 9-6 | 100 |
| 19 | M/R/9.2 | 95 | 2/3/4 | CONSERVATIVE | 12-16 | ORIF | YES | 2 SCREWS + 1 K-WIRE | 15 | 0-140 | 8-6 | 85 |
| 20 | M/R/3.1 | 7 | 2/3/4 | CONSERVATIVE | 10-12 | ORIF | NO | 5 K-WIRES | 10 | 0-140 | 8-5 | 65 |
| 21 | F/R/9.3 | 95 | 2/3/4 | SURGICAL | 7-8 | ORIF | NO | 1 SCREW + 1 K-WIRE | 20 | 0-140 | 5-4 | 65 |
| 22 | F/R/5.4 | 1.5 | 2/3/4 | CONSERVATIVE | 10-14 | ORIF | NO | 3 K-WIRES | NA | 45-90 | 3-0 | 30 |
| 23 | M//L7.1 | 8 | 1/3/5 | SURGICAL | 10-13 | ORIF | NO | 1 SCREW | 0 | 20-130 | 6-4 | 50 |
| 24 | M/R/4.4 | 1.5 | 2/3/4 | CONSERVATIVE | 5-7 | ORIF | NO | 1 SCREW + 1 K-WIRE | NA | 60-100 | 2-1 | 10 |
| 25 | M/R/14.5 | 152 | 2/3/4 | CONSERVATIVE | 18-19 | CO + ATUN | NO | PLATE | 33 | 0-140 | 3-3 | 65 |
| 26 | F/R/12.8 | 136 | 2/3/4 | CONSERVATIVE | 11-11 | CO + ATUN | NO | PLATE | 45 | 0-140 | 3-3 | 60 |
| 27 | M/R/14.7 | 92 | 2/2/3 | SURGICAL | 7-10 | CO + ATUN | NO | PLATE | 21 | 15-140 | 3-2 | 65 |

**Table S1**: *Baseline clinical, radiographic and surgical data of the patients included in the study*. M: male, F: female, R: right, L: left; ISF: In Situ Fixation; ORIF: Open Reduction and Internal Fixation; CO: Corrective Osteotomy; ATUN: Anterior Trasposition of Ulnar Nerve; MEPS: Mayo Elbow Performance Score.
