# Supplementary material for: Neglected Fractures of the Lateral Humeral Condyle in Children; Which Treatment for Which Condition?
Source: Children (Basel). 2021 Jan 18;8(1):56. doi: 10.3390/children8010056 (PMC7830377; doi:10.3390/children8010056)
Supplement: Supplementary file 1 [file children-08-00056-s001.zip › table 2 suppl..docx]

| **PATIENT** | **COMPLICATIONS** | **CDS** | **FOLLOW-UP (YEARS)** | **TIME TO UNION (MONTHS)*** | **CARRYING-ANGLE (degrees)** | **FLEXION-EXTENSION (degrees)** | **DHILLON SCORE OVERALL-FUNCTION** | **MEPS** |
| --- | --- | --- | --- | --- | --- | --- | --- | --- |
| 1 | DELAYED UNION  FISHTAIL DEFORMITY  BONY PROMINENCE | 2 | 7 | 13 | 7 | 5-140 | 9-6 | 95 |
| 2 | DELAYED UNION  BONY PROMINENCE | 2 | 2 | 4 | 7 | 0-140 | 9-6 | 100 |
| 3 | DELAYED UNION  BONY PROMINENCE | 2 | 2 | 3.5 | 10 | 5-135 | 8-5 | 100 |
| 4 | DELAYED UNION | 2 | 10 | 7.5 | 7 | 0-140 | 9-6 | 100 |
| 5 | DELAYED UNION | 2 | 13 | 4 |  | 0-140 | 9-6 | 100 |
| 6 | SEVERE VALGUS  FISHTAIL DEFORMITY  REOPERATION^1^ | 3 | 7 | - | 50 | 0-145 | 6-6 | 95 |
| 7 | PHYSEAL ARREST | 2 | 15 | 1 | 15 | 30-140 | 7-5 | 100 |
| 8 | NONE | 0 | 8 | 1 | 4 | 0-145 | 8-6 | 100 |
| 9 | NONE | 0 | 8 | 1 | 8 | 0-145 | 9-6 | 100 |
| 10 | BONY PROMINENCE | 1 | 4 | 1 | -10 | 0-145 | 7-6 | 100 |
| 11 | RECURRENT NONUNION  REOPERATION  REFRACTURE^2^ | 4 | 9 | 10 | -5 | 0-145 | 7-6 | 100 |
| 12 | NONE | 0 | 7 | 2 | 7 | 0-140 | 9-6 | 100 |
| 13 | DELAYED UNION | 2 | 2 | 6 |  | 0-140 | 9-6 | 100 |
| 14 | BONY PROMINENCE  FISHTAIL DEFORMITY | 1 | 12 | 1 | 10 | 10-140 | 8-5 | 100 |
| 15 | DELAYED UNION  BONY PROMINENCE  REFRACTURE^3^ | 2 | 11 | 9 | 5 | 0-145 | 8-6 | 100 |
| 16 | DELAYED UNION  VARUS PERSISTENCE  LATERAL INSTABILITY | 3 | 9 | 10 | -20 | 0-145 | 6-6 | 95 |
| 17 | INTRA-OPERATIVE WIRE RUPTURE WITHOUT CONSEQUENCES  HYPERTROFIC SCAR | 1 | 7 | 1 | 0 | 0-145 | 8-6 | 100 |
| 18 | NONE | 0 | 7 | 1 | 7 | 0-145 | 9-6 | 100 |
| 19 | DELAYED UNION  FISHTAIL DEFORMITY | 2 | 6 | 25 | 8 | 0-130 | 8-5 | 100 |
| 20 | NONE | 0 | 16 | 1 | 10 | 0-140 | 9-6 | 100 |
| 21 | RESIDUAL VALGUS | 3 | 3 | 3 | 20 | 0-140 | 7-6 | 95 |
| 22 | AVN | 4 | 10 | 1 | 7 | 30-120 | 6-3 | 80 |
| 23 | DELAYED UNION  BONY PROMINENCE | 2 | 2 | 8 | 5 | 15-135 | 7-5 | 100 |
| 24 | ELBOW STIFFNESS  MALUNION  REOPERATION^4^ | 3 | 12 | 1 | 7 | 30-110 | 9-6 | 75 |
| 25 | NONE | 0 | 3 | - | 10 | 0-145 | 9-6 | 100 |
| 26 | VALGUS RECURRENCE WITH RECURRENT ULNAR NERVE PALSY | 4 | 2 | - | 37 | 0-145 | 3-3 | 60 |
| 27 | NONE 0 | 0 | 2 | - | 8 | 0-140 | 9-6 | 100 |

**Table S2**: *Outcome data of the patients included in the study*. CDS: Clavien-Dindo-Sink classification system. MEPS: Mayo Elbow Performance Score. * In conservative cases, the time to union was calculated from injury to complete bone healing. In patients undergoing corrective osteotomy the time to union was not evaluated, since during operation the nonunion site was not repaired. In all cases the osteotomy site healed uneventfully at 30 days of follow-up. ^1^: This child presented three months after injury with a severely displaced LHC fracture (figure 2). ORIF was recommended but parents refused treatment. The child presented 5.5 years later with persistent nonunion and severe valgus elbow deformity. Corrective osteotomy was performed. ^2^: One child undergoing ISF with two K-Wires had no progression of healing within six months after surgery. The patient had further surgery with screw fixation and bone allograft. Then, the nonunion healed uneventfully four months later, but the child had another undisplaced LHC fracture 18 months after surgery, that healed satisfactorily with cast immobilization for 4 weeks. ^3^: undisplaced intercondylar fracture 9 years after treatment. Treated by 4-weeks cast immobilization. ^4^: reoperation for malunion with instability and pain 10 years later.
