# Supplementary material for: Neglected Fractures of the Lateral Humeral Condyle in Children; Which Treatment for Which Condition?
Source: Children (Basel). 2021 Jan 18;8(1):56. doi: 10.3390/children8010056 (PMC7830377; doi:10.3390/children8010056)
Supplement: Supplementary file 1 [file children-08-00056-s001.zip › table 3 supplementary.docx]

| **Correlations within baseline variables** | | |
| --- | --- | --- |
| **variables** | **Spearman’s r** | ***P*-value** |
| Age/injury duration | 0.45 | 0.019 |
| Injury duration/lateral displacement | 0.54 | 0.004 |
| Injury duration/medial displacement | 0.41 | 0.034 |
| Injury duration/baseline ROM | 0.72 | <0.0005 |
| Injury duration/baseline functional DhiS | 0.46 | 0.015 |
| Weiss/Song | 0.67 | <0.0005 |
| Weiss/lateral displacement | 0.53 | 0.004 |
| Weiss/medial displacement | 0.79 | <0.0005 |
| Song/ lateral displacement | 0.63 | <0.0005 |
| Song/medial displacement | 0.61 | 0.001 |
| Lateral displacement/medial displacement | 0.75 | <0.0005 |
| Lateral displacement/Baseline elbow carry angle | 0.62 | 0.003 |
| Baseline elbow carry angle/age | 0.55 | 0.011 |
| Lateral displacement/baseline ROM | 0.43 | 0.025 |
| Medial displacement/baseline ROM | 0.46 | 0.016 |

**Table S3**: *Correlations between baseline variables*. Only correlations with Spearman’s r>0.4 and p-value<0.05 were reported. ROM: range of motion. DhiS: Dhillon score.
